# Dietary Intake and Rural-Urban Migration in India: A Cross-Sectional Study

**DOI:** 10.1371/journal.pone.0014822

**Published:** 2011-06-22

**Authors:** Liza Bowen, Shah Ebrahim, Bianca De Stavola, Andy Ness, Sanjay Kinra, A.V. Bharathi, Dorairaj Prabhakaran, K. Srinath Reddy

**Affiliations:** 1 Department of Epidemiology and Population Health, London School of Hygiene and Tropical Medicine, London, United Kingdom; 2 South Asia Network for Chronic Disease, Public Health Foundation of India, New Delhi, India; 3 School of Oral and Dental Sciences, University of Bristol, Bristol, United Kingdom; 4 Department of Food Science and Nutrition, Indira Gandhi National Open University, New Delhi, India; 5 Centre for Chronic Disease Control, New Delhi, India; 6 Public Health Foundation of India, New Delhi, India; Pennington Biomedical Research Center, United States of America

## Abstract

**Background:**

Migration from rural areas of India contributes to urbanisation and lifestyle change, and dietary changes may increase the risk of obesity and chronic diseases. We tested the hypothesis that rural-to-urban migrants have different macronutrient and food group intake to rural non-migrants, and that migrants have a diet more similar to urban non-migrants.

**Methods and findings:**

The diets of migrants of rural origin, their rural dwelling sibs, and those of urban origin together with their urban dwelling sibs were assessed by an interviewer-administered semi-quantitative food frequency questionnaire. A total of 6,509 participants were included. Median energy intake in the rural, migrant and urban groups was 2731, 3078, and 3224 kcal respectively for men, and 2153, 2504, and 2644 kcal for women (p<0.001). A similar trend was seen for overall intake of fat, protein and carbohydrates (p<0.001), though differences in the proportion of energy from these nutrients were <2%. Migrant and urban participants reported up to 80% higher fruit and vegetable intake than rural participants (p<0.001), and up to 35% higher sugar intake (p<0.001). Meat and dairy intake were higher in migrant and urban participants than rural participants (p<0.001), but varied by region. Sibling-pair analyses confirmed these results. There was no evidence of associations with time in urban area.

**Conclusions:**

Rural to urban migration appears to be associated with both positive (higher fruit and vegetables intake) and negative (higher energy and fat intake) dietary changes. These changes may be of relevance to cardiovascular health and warrant public health interventions.

## Introduction

In India the disease burden is changing. Communicable diseases remain a major problem, but there is a rapid emergence of chronic disease, including obesity, diabetes, and cardiovascular disease (CVD); CVD now accounts for an estimated 27% of deaths in India [1]–[4]. Diet is an established risk factor for CVD [5]–[7], so it is important to study the changes that occur alongside urbanisation, increased economic prosperity, and globalisation[8]–[9].

Rural-urban migrants experience the environmental changes associated with urbanisation very rapidly, enabling epidemiologic transitions to be examined. Changes seen in migrants over relatively short time periods may therefore provide insights into the wider population health changes associated with urbanisation. Regional rural and urban studies have shown higher rates of chronic disease in urban than rural areas [10]–[13], and recent data on rural-urban migrants in India have shown that migration is associated with marked increases in obesity and diabetes [14]. As diet is an important risk factor for both obesity and diabetes, understanding the changes in dietary intake may provide clues to the causes of these increases in chronic conditions.

Among rural-urban migrants in Guatemala [15] and in China [16]–[17] reductions in energy intake, but increases in proportion of energy from fat, and rises in saturated fat and cholesterol intake were found compared with non-migrant rural counterparts. In addition the Guatemala study found higher fruit and vegetable consumption in migrants than rural participants. The Chinese study found higher sodium intake in migrants than rural participants, which was also seen in a migration study in Kenya [18]. International migration studies have found varied effects of migration on diet [19]–[30], often from a traditional to more western style of diet.

While there are no rural-urban migration studies from India, there are data on national trends in dietary intake and on rural urban comparisons. Consumption data from Food and Agricultural Organisation (FAO) food balance sheets from the last 40 years shows increase in available energy (from ∼2000 kcal/day to ∼2500 kcal/day), protein and fat, with the steepest rises in fat intake.[31]

The National Sample Survey Organisation (NSSO) conducts dietary intake surveys in rural and urban India, asking about household consumption over the past 30 days. The 2004-5 survey found total energy intake to be very similar in rural and urban areas (2047 kcal and 2020 kcal respectively), but fat intake was much higher in urban (48 g) compared with rural (36 g) areas. While the proportion of energy from cereals was higher in rural than urban people, the proportion of energy from most other food groups was higher in urban people, most notably milk products and oils and fats, but also fruit and vegetables.[32]

The objective of this analysis was therefore to measure the dietary differences associated with rural-urban migration to help explain increases in obesity and diabetes in urban India. Specifically, comparisons in macronutrient and food group intake between rural-urban migrants and both their rural and urban counterparts were made.

## Methods

### Study design and data collection

The Indian Migration Study (IMS) was set up to examine the effects of rural to urban migration on obesity and diabetes. The study was nested in a larger sentinel surveillance study of cardiovascular risk factors in industrial settings [33], and used a sibling-pair comparison design in which urban factory workers who had migrated from rural areas were recruited together with their rural-dwelling sibling who had not migrated. Details of the design and major findings have been reported elsewhere.[14]

Briefly, the study was based in factories in four Indian cities (Lucknow - Hindustan Aeronautics Ltd; Nagpur - Indorama Synthetics Ltd, Hyderabad - Bharat Heavy Electricals Ltd and Bangalore – Hindustan Machine Tools Ltd) situated in the north, centre and south of the country. Factory workers and their co-resident spouses were recruited if they were rural-urban migrants, using employer records as the sampling frame. Each participant was asked to invite one non-migrant full sibling of the same sex and closest to them in age still residing in their rural place of origin. A 25% random sample of urban non-migrant factory workers and spouses was invited to participate in the study, and also asked to invite a sib who resided in the same city but did not work in the factory. Information sheets were translated into local languages and explained to participants by trained interviewers and signed (or thumb print used if illiterate) to indicate informed consent. Ethics committee approval was obtained from the All India Institute of Medical Sciences Ethics Committee. Field work began in March 2005 and was completed by December 2007.

Diet was assessed by an interviewer-administered semi-quantitative food frequency questionnaire (FFQ). The questionnaire assessed portion size and frequency of intake of 184 commonly consumed food items, asking about consumption over the last year. A standard portion size was assigned to each food (e.g. tablespoon, ladel, bowl), and participants were shown examples of these vessels and asked to report portions consumed as multiples of this. Frequency was recorded as daily, weekly, monthly, yearly/never. A single FFQ was used to cover the four regions of the study. Nutrient databases were developed for the study by collecting recipes from participants in rural and urban areas of each region, and using Indian food composition tables to calculate nutrient content of each recipe [34]. Where nutrient values were unavailable from the Indian food composition tables (e.g. for foods such as manufactured ‘western’ snacks and sweetened drinks), the United States Department of Agriculture nutrient database (USDA, Release No. 14) [35] or McCance and Widdowsons Composition of Foods were used [36]. Because of variation in food preparation, region, rural/urban, and cooking oil specific databases were used for calculation of average daily dietary intake. Energy, carbohydrate, fat, saturated fat, protein, as well as proportion of energy from carbohydrate, fat and protein, and energy density, were considered. The recipes were also used to generate databases of the food group composition of each food item, and used to calculate average daily food group intake. For this analysis the following food groups were considered: fruit; vegetables (including vegetables added to preparations, and salads); legumes (pulses, lentils, whole gram preparations); sugar (sugar and jaggery used in preparations and added to beverages); meat (including meat added to mixed dishes); fish; and dairy (including dairy products added to beverages and preparations).

To measure socio-economic position, a subset of 14 of 29 questions were used from the Standard of Living Index (SLI), a household level asset based scale devised for Indian surveys,[37] selecting those believed to be most informative for the study population, and weighting them to give a maximum score of 38. Weights of items for the SLI developed by the International Institute of Population Sciences in India, and based on *a priori* knowledge about the relative significance of the items, were used in these analyses.

Analyses of IMS data to date have shown large differences in chronic disease profile. For example, BMI in rural, migrant and urban men was 21.9, 24.0 and 24.3 respectively (p<0.0001) and in women 22.5, 25.2, and 25.9 respectively(p<0.0001)[14]. Initial analyses of the dietary data also showed that consumption was mainly of traditional foods in rural, migrant, and urban groups, and that there were low levels of western foods consumption [38].

### Statistical methods

Rural-urban migrants, their rural siblings, and urban non-migrants and their urban siblings were included in analyses. Distributions of macronutrient intake were checked for outliers. Median values and lower and upper quartiles were calculated (because of the skewed distribution of some variables) for energy intake, macronutrient intake (protein, fat, saturated fat, and carbohydrate, and percent energy from each of these), and energy-density, by sex and migration status. Nutrients were transformed to the natural log scale and statistical tests for the significance of the trend in mean values across rural, migrant, and urban groups were calculated using linear regression models and Wald test statistics, adjusted for age and factory site. Analyses were carried out separately in men and women, as we anticipated that there may be gender differences in the effect of migration, and also because of the statistical dependency between husbands and wives produced by the study design. Robust standard errors were used to account for clustering in sibling pairs. Similar analyses were conducted to examine variation in food groups by migration status. Vegetarianism was assessed by whether the participant reported consumption of any meat or fish in the FFQ. Differences in the proportion of vegetarians in rural, migrant and urban groups were assessed via Wald test statistics from a logistic regression model, adjusting for age, factory, and again accounting for clustering in sibling pairs by calculating robust standard errors. For meat and fish, differences in intake were considered for participants who were non-vegetarians only.

Further analyses examining differences (suitably transformed) in intake between migrant and non-migrant siblings, taking advantage of the sibling-pair design, were also conducted as this comparison provides a high level of control for known and unknown genetic and early life confounders. Only rural-urban migrant pairs were included in these analyses as urban non-migrant sib-pairs were not informative for assessing migration effects. Because not all siblings were matched on sex, sex-specific z-scores of intake were calculated using the rural distribution as reference. Within pair differences in z-scores for energy, macronutrients, energy density, and food groups were considered and modelled using linear regression. Although the sibling closest in age was recruited, differences in age were inevitable so the within-pair difference in age was considered as a potential confounder. Factory was not controlled for because it did not vary within pairs. The estimated intercept in these models is to be interpreted as the estimated mean difference in z-scores between same-age siblings and therefore as the age-adjusted effect of migration. Mean differences and 95% confidence intervals were graphed to aid interpretation. Differences in energy-adjusted intake were calculated using the residuals method [39], calculating z-scores of residuals in each sibling, and taking the difference of these scores. The additional effect of the amount of time the migrant sibling had spent in urban areas (with categories: <10 years or 10+ years) was also studied by extending each of the linear regression models used to study the macronutrients and the food groups, with significance assessed via Wald tests. Similarly, effect differences by factory were studied by including factory indicators in the models.

All analyses were conducted using STATA 11 statistical software (StataCorp. 2009. *Stata Statistical Software: Release 11*. College Station, TX: StataCorp LP).

## Results

### Response

**Figure 1:**
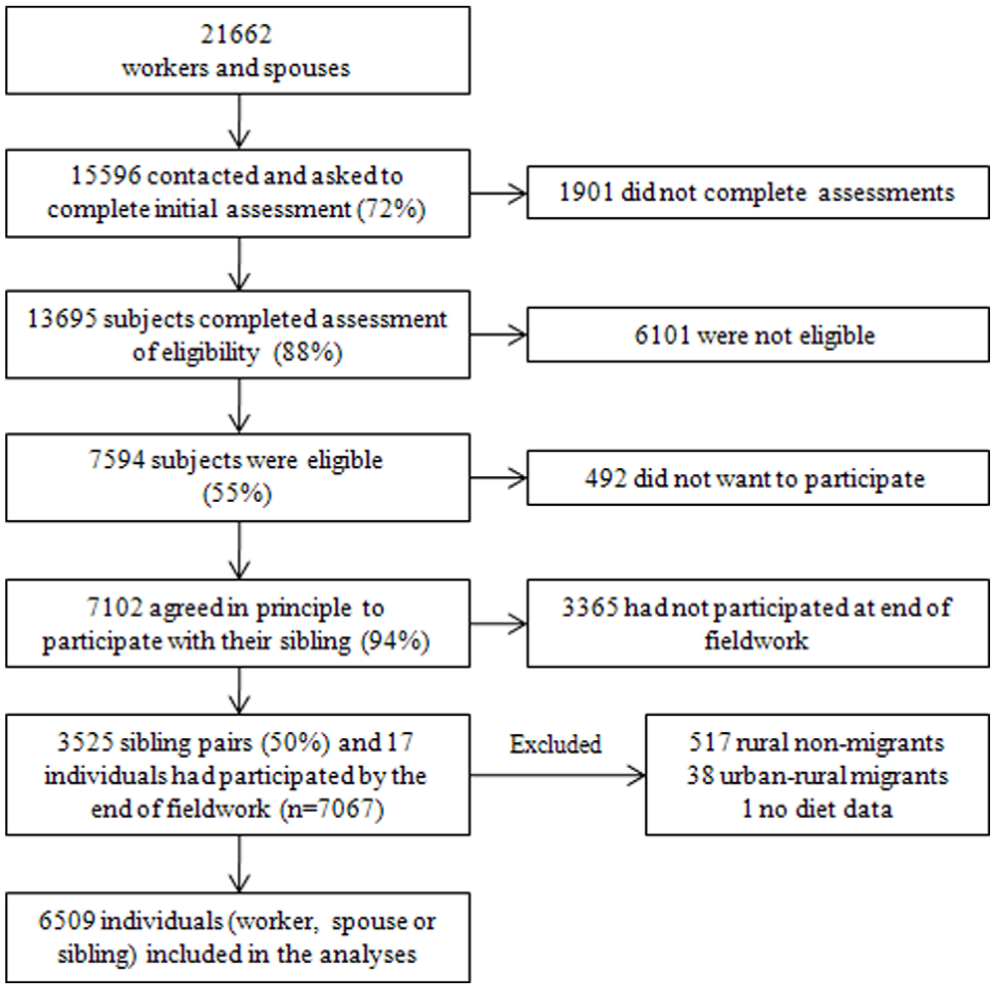
Response rate in the Indian Migration Study.

Figure 1 shows response at different points of the study. Employee records indicated that 21,662 workers and spouses at the four factories were available for study, with 15,596 (72%) identified as still working in the factory and contacted. A total of 13,695 (88%) individuals completed the assessment of eligibility for the study; 7,594 (55%) of them were eligible for inclusion as they were a migrant with a rural dwelling sib or were selected as part of a random 25% sample of urban non-migrants. Most of the eligible subjects, 7,102 (94%), agreed in principle to complete the clinical examination with their sibling but only half, 3525 (50%), came to clinics along with their siblings, while 17 individuals came without siblings, giving a total of 7067 individuals. Factory workers who lived in rural areas and commuted to work (n = 519) and urban-rural migrants (n = 38) were excluded from these analyses. One individual was excluded because no dietary information was available.

### Demographic and social characteristics

A total of 6509 participants were included in analysis, of whom 2111 were rural, 2112 were migrants, and 2286 were urban dwellers (Table 1). Of the total, 1964 (30%) were sampled from Lucknow, 1417 (22%) from Nagpur, 1727 (27%) from Hyderabad, and 1401 (22%) from Bangalore. The mean ages of men and women were 42 and 40 years respectively. Socio-economic position was similar in migrant and urban participants, but considerably lower in rural participants. Over 80% of participants were married, but the proportion married was lowest in rural men (81%) and women (76%). Manual work was less common in females (10%) than males (58%), and was more common in rural than migrant and urban participants. By site, the proportion of men doing manual work in the factories was: Lucknow (27%), Nagpur (97%), Hyderabad (75%), and Bangalore (35%). The proportion of participants with secondary education was lower in rural than migrant and urban groups. Over 90% of the sample was of Hindu religion, although the proportion was slightly lower in the urban non-migrant group (85% in men, 87% in women).

**Table 1:** Study population characteristics by sex and migration status. Indian migration study, 2005–2007.

	Men				Women			
	Rural	Migrant	Urban	Total	Rural	Migrant	Urban	Total
Number	1459	1127	1201	3787	652	985	1085	2722
Age, Mean (SD)	39.6(11.6)	44.7(8.6)	41.5(10.0)	41.8(10.5)	41.4(11.4)	39.6(8.7)	39.7(9.6)	40.1(9.8)
(Min; Max)	(17.0,76.0)	(25.0,65.0)	(18.0,70.0)	(17.0,76.0)	(17.0,70.0)	(19.0,59.0)	(18.0,68.0)	(17.0,70.0)
Standard of living index, Mean (SD)	17.3(6.7)	25.1(4.3)	24.5(5.0)	21.9(6.6)	16.3(6.8)	24.7(4.2)	24.7(5.2)	22.7(6.4)
(Min; Max)	(2.0,38.0)	(11.0,36.0)	(6.0,38.0)	(2.0,38.0)	(2.0,34.0)	(11.0,34.0)	(4.0,38.0)	(2.0,38.0)
Married, N (%)	1184(81.2)	1108(98.3)	1015(84.5)	3307(87.3)	495(75.9)	964(97.9)	952(87.7)	2411(88.6)
Manual worker, N (%)	1001(68.6)	680(60.3)	524(43.6)	2205(58.2)	118(18.1)	48(4.9)	107(9.9)	273(10.0)
Secondary edcuation, N (%)	1053(72.2)	1073(95.2)	1119(93.2)	3245(85.7)	253(38.8)	546(55.4)	947(87.3)	1746(64.1)
Hindu religion, N (%)	1381(94.7)	1066(94.6)	1025(85.3)	3472(91.7)	594(91.1)	905(91.9)	940(86.6)	2439(89.6)

### Dietary differences

Median reported energy intake was lowest in rural participants, higher in migrants and highest in urban participants, in both men and women (2731 kcal, 3084 kcal, 3225 kcal in men, p<0.001) (Table 2). The same pattern was seen for energy-density, fat, saturated fat, carbohydrates, and protein (all p<0.001). Differences between these groups in proportion of energy from macronutrients were small but migrant and urban men had a higher proportion of energy from fat, saturated fat and protein than rural men, and a lower proportion from carbohydrates. The same pattern was seen in women, except migrant women had similar proportions of energy from saturated fat as rural women.

**Table 2:** Energy intake, macronutrient intake, and energy density of diet, by sex and migration status.

	Men				Women			
	Rural	Migrant	Urban	p-trend†	Rural	Migrant	Urban	p-trend†
Energy (kcal)	2731(2096, 3543)	3084(2493, 3794)	3225(2659, 3893)	<0.001	2151(1716, 2723)	2502(2111, 3071)	2644(2208, 3203)	<0.001
Energy density (kcal/g)	1.32(1.14, 1.50)	1.36(1.17, 1.60)	1.39(1.19, 1.55)	<0.001	1.23(1.11, 1.40)	1.30(1.15, 1.54)	1.34(1.17, 1.51)	<0.001
Fat (g)	73( 54, 99)	86( 67, 116)	92( 71, 118)	<0.001	59( 43, 77)	72( 57, 93)	76( 61, 99)	<0.001
(% energy)	24.8(20.7, 28.5)	25.7(22.6, 28.9)	25.9(22.9, 28.9)	<0.001	24.9(21.0, 28.4)	25.8(23.0, 29.0)	26.4(23.4, 29.2)	<0.001
Saturated fat (g)	22( 16, 32)	25( 19, 33)	28( 21, 37)	<0.001	18( 12, 26)	21( 15, 27)	23( 17, 30)	<0.001
(% energy)	7.4( 5.7, 9.3)	7.4( 6.1, 8.6)	7.7( 6.4, 9.4)	0.651	7.3( 5.6, 9.4)	7.3( 6.1, 8.8)	7.9( 6.5, 9.4)	0.024
Carbohydrate (g)	434( 334, 568)	483( 390, 596)	501( 415, 604)	<0.001	344( 275, 443)	395( 331, 479)	413( 345, 495)	<0.001
(% energy)	63.3(59.4, 68.2)	63.0(59.5, 65.9)	62.4(59.3, 65.3)	<0.001	63.9(60.0, 68.5)	63.1(60.1, 65.9)	62.6(59.6, 65.2)	<0.001
Protein (g)	78( 59, 100)	89( 70, 107)	95( 77, 113)	<0.001	58( 45, 74)	71( 59, 86)	76( 61, 91)	<0.001
(% energy)	11.3(10.2, 12.3)	11.3(10.6, 12.2)	11.5(10.7, 12.5)	<0.001	10.7( 9.9, 11.8)	11.2(10.4, 12.1)	11.3(10.4, 12.2)	0.007

† p-values from Wald tests for trend adjusting for age and factory site and allowing for clustering of siblings.

For food groups, the same pattern of greater consumption in migrant and urban participants than rural participants was seen. For fruit intake, migrants had a similar median intake to the urban group (148 g and 146 g in men), which was higher than the rural group (101 g in men) (p<0.001). For vegetables, sugars, and dairy products there was a trend of increased consumption from rural to migrant to urban (e.g. median sugar consumption in women was 26 g, 30 g, and 35 g in rural, migrant, and urban groups respectively, p<0.001).

The proportion of vegetarians was 34% (n = 713), 35% (n = 734) and 38% (n = 860) in rural, migrant, and urban groups respectively (p = 0.16 in men, p = 0.15 in women). Among non-vegetarians there was a trend of increase in median meat consumption across rural, migrant, and urban groups (19 g, 25 g, 28 g in men respectively, p<0.001). No evidence of differences in fish intake by migration status was found, although levels of fish consumption were very low (p = 0.32 in men, p = 0.59 in women).

### Sibling pair analyses

**Figure 2:**
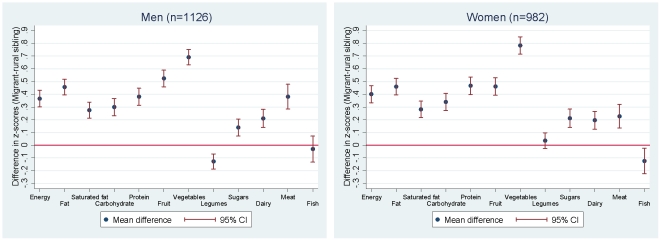
Sibling pair differences in z-scores† for nutrients and food group intake (migrant – rural sibling), adjusted for differences in age. † z-scores were generated by log-transformation of the original food intake, followed by sex standardisation based on the sex-specific distribution of the rural participants.

Figure 2 addresses similar questions to tables 2 and 3, but used the sib-pair design to make comparisons between siblings on a z-score scale. The results were entirely consistent with the aggregate analysis of rural versus migrant findings. The use of z-scores allowed comparison of the magnitude of change in the different diet variables, and showed that the greatest differences between the urban and rural dwelling sib-pairs was in vegetables and fruit consumption.

**Table 3:** Food group intake, by sex and migration status.

	Men				Women			
	Rural	Migrant	Urban	p-trend†	Rural	Migrant	Urban	p-trend†
Fruit (g)	101( 56, 178)	148( 90, 234)	146( 82, 242)	<0.001	89( 48, 162)	144( 81, 225)	145( 86, 229)	<0.001
Vegetables (g)	201(132, 287)	294(210, 412)	334(233, 430)	<0.001	160(112, 231)	249(176, 351)	288(209, 388)	<0.001
Legumes (g)	59( 34, 91)	52( 32, 75)	60( 41, 87)	0.170	41( 26, 70)	43( 29, 65)	51( 36, 71)	0.837
Sugars (g)	34( 22, 49)	34( 24, 49)	43( 30, 60)	<0.001	26( 16, 37)	30( 21, 42)	35( 25, 48)	<0.001
Dairy (g)	303(179, 471)	351(234, 487)	381(254, 540)	<0.001	249(148, 384)	287(191, 426)	328(209, 471)	<0.001
Meat (g)*	19( 9, 36)	25( 12, 46)	28( 13, 50)	<0.001	16( 7, 31)	23( 10, 40)	20( 10, 39)	<0.001
Fish (g)*	7( 2, 16)	6( 2, 12)	6( 2, 15)	0.324	6( 2, 14)	5( 2, 13)	4( 2, 12)	0.591

†p-values from Wald tests for trend adjusting for age and factory site and allowing for clustering of siblings

*for meat and fish, median values are given for consumers (i.e. non-vegetarians) only.

**Figure 3:**
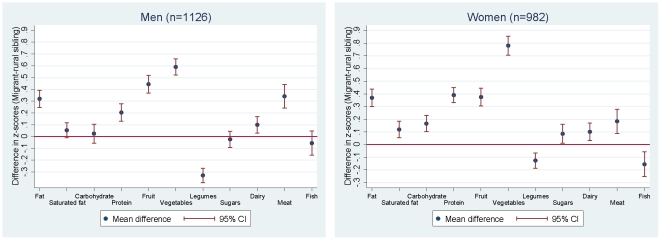
Sibling pair differences in z-scores† for energy-adjusted nutrients and food group intake (migrant – rural sibling), adjusted for differences in age. † z-scores were generated by log-transformation of the original food intake, followed by sex standardisation based on the sex-specific distribution of the rural participants.

When comparing the sibling differences in migrants of <10 years duration to those of 10+ years duration, no difference in effect was seen for any of the dietary variables considered (Table S1). Energy-adjusted results are also shown (Figure 3), and can be interpreted as differences in the composition of the diet, i.e. difference in intake relative to total energy intake. In women, the same patterns of difference between rural and migrant siblings was seen, with slight attenuation of effect. In men, there was attenuation of effect, and for saturated fat, carbohydrate, sugars and fat, there was no evidence of difference between rural and migrant. So although the migrant men were consuming higher overall levels of saturated fat, carbohydrate, sugars and fat, as a proportion of their total energy intake there was no evidence of difference from rural siblings.

### Variation between factories

The overall prevalence of vegetarianism varied between factories. The prevalence was 15% in Hyderabad, 27% in Nagpur, 31% in Bangalore, and 56% in Lucknow. Median meat consumption was higher in the southern factories (Hyderabad 29 g and Bangalore 29 g) than the northern factories (Lucknow 12 g and Nagpur 14 g). Median dairy intake was highest in Lucknow (406 g), lowest in Nagpur (217 g), and intermediate in Hyderabad and Bangalore (298 g, 363 g). When sib-pair differences in z-scores of nutrient and food group intake were considered by factory there was variation in the migration effect (p<0.001 in heterogeneity tests for all variables) (Figure S1). The most notable differences were for Bangalore, where energy, saturated fat, carbohydrate, protein, and meat intake were all lower in the migrant than the urban sibling, and legumes intake was higher in the migrant than rural sibling, contrary to what was seen in the other three factories. In Nagpur migrants had lower sugar intake than their rural siblings, contrary to the higher intake seen in other factories. In all factories, fat, fruit and vegetable, and dairy intake were higher in the migrant than rural siblings.

## Discussion

This analysis found that migration from rural to urban areas was associated with higher energy intake and consumption of most macronutrients and food groups. Only small differences in relative intake of macronutrients were seen: the proportion of energy from protein and fat was a little higher in migrant and urban than rural groups (<2% difference), and the proportion from carbohydrate a little higher in rural groups. For some dietary factors (fruit, meat) migrants had reached the higher levels of consumption of urban participants, while for others there was a trend of increasing intake across rural, migrant, and urban groups (vegetables, sugars, dairy, and macronutrients).

The sibling pair design used was beneficial because it provided a high level of control for potential confounding, and consistency between analyses in group and sib-pairs makes the findings more robust. However, the requirement of siblings to travel to the factories to participate in the study created a high responder burden, and the response rate of 50% could have introduced selection bias, although previous analyses comparing those who took part with those who did not take part found no strong evidence of difference in health status[14]. Response bias would exaggerate differences seen in dietary intake if there was differential non-response whereby rural participants with the most different diets to their migrant siblings were more likely to participate. It is difficult to assess the likelihood of such non-response, but it is unlikely to explain the substantial differences observed between migrant and rural siblings.

Measurement of diet is prone to error. An FFQ was the only feasible method for measuring intake given that the study design required rural participants to travel on the days prior to assessment. The validity study found that the FFQ overestimated intake by 409 kcal compared to an average taken from three administered 24 hr recalls; for most nutrients and food groups error was non-differential between the rural and urban participants, however there was evidence that median overestimation of fat and vegetable intake was higher among urban than rural people (18 g vs 11 g respectively for fat and 75 g vs 33 g for vegetable intake). This difference in reporting could explain some (but unlikely all) of the large differences in vegetable intake found in the analyses presented here. Overestimation of energy intake is a common finding in FFQs, and was also found in validation studies for region-specific FFQs conducted in India, which showed a similar level of validity for the macronutrients considered in this paper (validity of food group intake is rarely reported)[40]–[43]. From these results it is important to note that the data are acceptable for comparing between groups but that the intake values should not be considered an accurate reflection of absolute levels of intake. The limitation of using the Indian food tables that were constructed in 1971 should also be noted- there may have been some changes in content of foods since that time, and while tables are currently being updated by the National Institute of Nutrition, the 1971 tables are the best available at this time. For packaged foods, USDA tables should have provided a reasonable estimation of nutrient content for these more recently available foods.

The participants were sampled from factories and were therefore better off and more likely to be in stable employment than the Indian population in general. As well as being wealthier than other streams of migrants, the IMS migrant group worked in factories that provide housing and canteen facilities, and this may have accelerated the process of acculturation to urban lifestyle and diets. The complex nature of migration as an exposure makes it difficult to generalize our results to other groups of migrants. In addition most of the participants were middle aged (mean age 41), and there were insufficient numbers to study how migration effects might differ at younger ages. Many of the convenience and fast food advertising campaigns are targeted at younger populations and there may be consumption of higher fat and energy density diets that were not captured here. In addition, because most of the participants had been in the urban areas for a long time, it was not possible to discern early migration effects and speed of adoption of urban diets within the first 10 years. It would be useful in future studies of migrant populations to consider migration effects in younger and more recent migrant populations.

Migration studies in Guatemala and China (the only two studies of internal migration that measure dietary differences in a comparable way) found lower energy intake in migrant than rural people whereas here the opposite was found [15]–[16]. Differences in the rural populations sampled may be the explanation; the Guatemala and China studies reported rural populations that were predominantly manual workers requiring higher energy intake, whereas in the rural IMS group 42% of men and 90% of women were in non-manual occupations. The higher proportion of energy from fat found was consistent with these other rural-urban migration studies, as was the increase in saturated fat intake, fruit and vegetables, and meat intake.

Dietary intake is of interest because of its association with chronic diseases such as CVD and diabetes. Some of the differences seen are potentially positive in terms of chronic disease risk (e.g. increased fruit and vegetable intake[44]–[46]), while others are associated with increased risk of chronic disease (e.g. increased saturated fat intake [47]). While the results should be considered with caution because FFQs are not good at estimating absolute intake, it is interesting to note that the diets of rural, migrant, and urban groups are all much lower in fat and saturated fat compared to developed country populations, and to relevant guidelines[48]–[49]. So, even given the increases in fat intake compared with rural areas, the urban and migrant diet composition can still be considered relatively healthy.

The findings should be interpreted in the context of the higher obesity levels found in migrants compared with rural participants in previous analyses [14]. Obesity is the result of an imbalance between energy intake and energy output. The higher energy intake in migrants must be contributing to weight gain, given that previous analyses found lower levels of activity in the migrant and urban than rural people, suggesting that the energy requirements of the migrant and urban subjects should be even lower than the rural participants [14]. While there is error in absolute intakes calculated from FFQs, the magnitude of difference in energy intake between rural and migrant participants (∼300 kcal per day) is in the region of the 370 kcal per day that was recently predicted to result in changes in BMI from 23 to 29 over the course of 28 years in young women [50].

There was higher sugar intake in migrants than rural people. There is little evidence linking sugar consumption to obesity, diabetes and CVD [51]–[52], but this may be of interest to study further in Indian populations, where there is a different dietary profile to the western settings, and a rising burden of chronic disease [53].

Higher meat and dairy consumption could be a concern because of the high levels of saturated fat that they contain. However, despite increases, actual consumption remains low (and far below recommended as well as observed western levels), and some increase in animal products could be beneficial in increasing the amount of protein in the diet[54]. In addition, with the increase in low fat dairy options there has been some suggestion that there may be some positive effects of dairy consumption[55], and given the overall low intakes of fat and saturated fat, negative health effects of animal product intake are not a primary concern.

To develop useful policy suggestions from what we have seen of dietary changes after migration, qualitative studies would be beneficial, looking in greater depth at the reasons and modifying factors of both the positive and negative changes to diet associated with migration to urban areas.

The composition of the diets of migrants in this study is relatively healthy despite unhealthy trends such as higher fat intake than the rural population, but the higher energy intake must be contributing to the higher prevalence of overweight. This overall consumption excess is the main concern, and should be tackled while ensuring that the beneficial higher levels of fruit and vegetables remain easily attainable.

## Supporting Information

Figure S1 Sibling pair differences in z-scores† for nutrients and food group intake (migrant - rural sibling), adjusted for differences in age, by factory. †-scores were generated by log-transformation of the original food intake, followed by standardisation based on the sex-specific distribution of the rural participants.
(0.05 MB TIF)

Table S1 Differences in z-scores† between migrant and sibling by time in urban areas (<10years and 10+ years), adjusted for age of the migrant, age difference between siblings, and factory.
(0.05 MB DOC)
