# Supplementary figures and images for: Dietary Intake and Rural-Urban Migration in India: A Cross-Sectional Study
Source: PLoS One. 2011 Jun 22;6(6):e14822. doi: 10.1371/journal.pone.0014822 (PMC3120774; doi:10.1371/journal.pone.0014822)

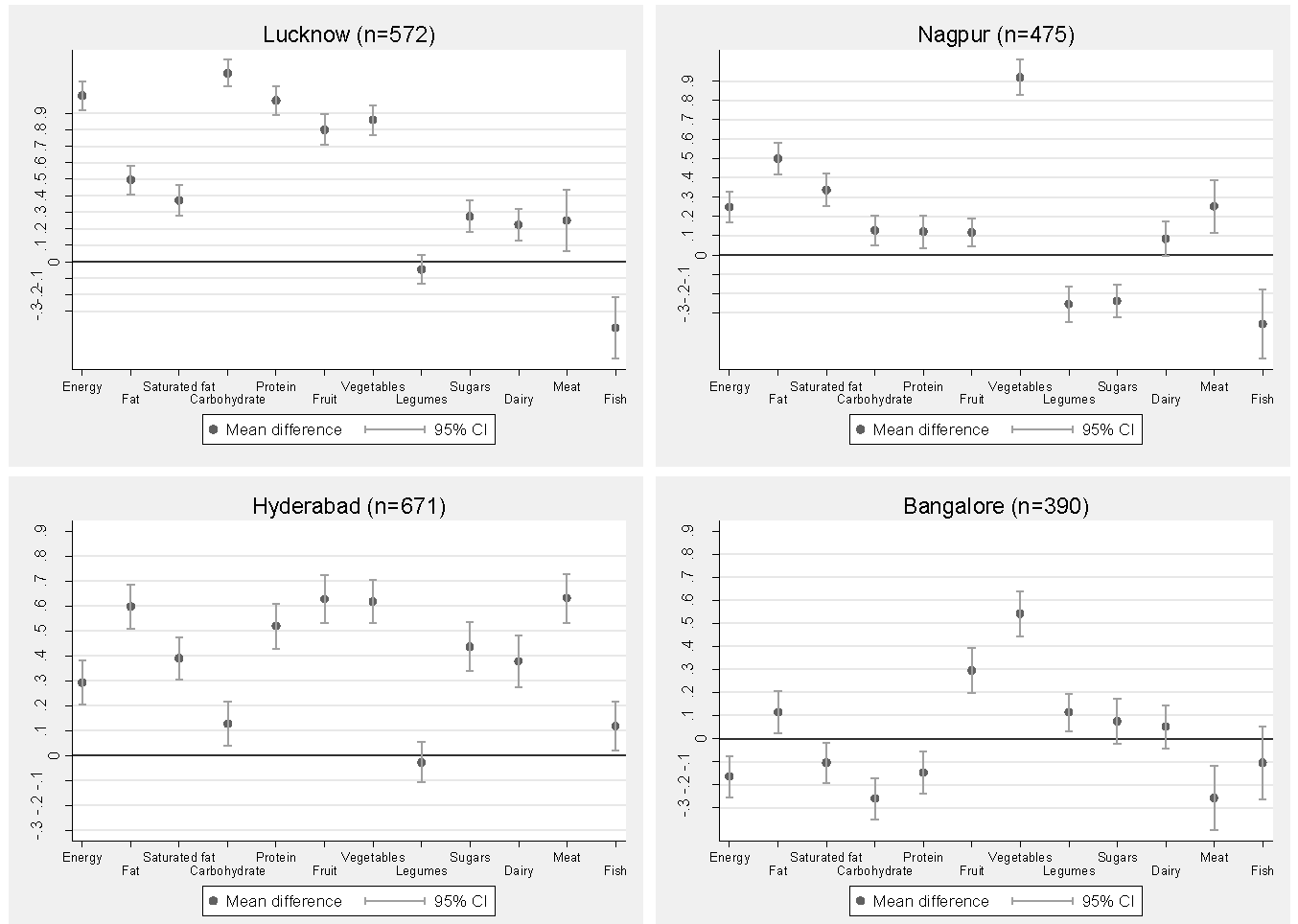

Supplement: Figure S1 — Sibling pair differences in z-scores† for nutrients and food group intake (migrant - rural sibling), adjusted for differences in age, by factory. †-scores were generated by log-transformation of the original food intake, followed by standardisation based on the sex-specific distribution of the rural participants. (0.05 MB TIF) [file pone.0014822.s001.tif]
