# Supplementary material for: Dietary Intake and Rural-Urban Migration in India: A Cross-Sectional Study
Source: PLoS One. 2011 Jun 22;6(6):e14822. doi: 10.1371/journal.pone.0014822 (PMC3120774; doi:10.1371/journal.pone.0014822)
Supplement: Table S1 — Differences in z-scores† between migrant and sibling by time in urban areas (<10years and 10+ years), adjusted for age of the migrant, age difference between siblings, and factory. (0.05 MB DOC) [file pone.0014822.s002.doc]

**Online Supplemental Material: Tables**

Table S1: Differences in z-scores† between migrant and sibling by time in urban areas (<10years and 10+ years), adjusted for age of the migrant, age difference between siblings, and factory

|  | Men |  |  | Women |  |  |
| --- | --- | --- | --- | --- | --- | --- |
|  | <10 years | 10+ years | p-value* | <10 years | 10+ years | p-value* |
| Energy | 0.60(0.92) | 0.32(1.10) | 0.083 | 0.25(0.85) | 0.43(1.07) | 0.087 |
| Energy density | 0.26(0.66) | 0.24(0.85) | 0.973 | 0.39(0.82) | 0.36(0.89) | 0.585 |
| Fat | 0.76(0.95) | 0.40(1.04) | 0.086 | 0.47(0.88) | 0.44(1.04) | 0.160 |
| Saturated fat | 0.57(0.89) | 0.23(1.05) | 0.129 | 0.29(0.95) | 0.26(1.01) | 0.139 |
| Carbohydrate | 0.46(0.91) | 0.26(1.14) | 0.156 | 0.13(0.84) | 0.39(1.09) | 0.112 |
| Protein | 0.56(1.07) | 0.34(1.12) | 0.083 | 0.16(0.89) | 0.54(1.08) | 0.173 |
| Fruit | 0.41(0.92) | 0.50(1.10) | 0.430 | 0.26(0.89) | 0.47(1.09) | 0.031 |
| Vegetables | 0.96(1.12) | 0.67(1.01) | 0.199 | 0.98(1.01) | 0.73(1.06) | 0.559 |
| Legumes | -0.00(0.85) | -0.15(0.99) | 0.251 | -0.14(1.05) | 0.07(0.95) | 0.468 |
| Sugars | -0.01(1.04) | 0.12(1.12) | 0.911 | -0.07(1.02) | 0.26(1.14) | 0.315 |
| Dairy | 0.31(1.02) | 0.20(1.18) | 0.503 | 0.08(1.11) | 0.22(1.08) | 0.498 |
| Meat | 0.46(1.27) | 0.36(1.21) | 0.827 | 0.21(1.10) | 0.22(1.09) | 0.048 |
| Fish | 0.09(1.06) | -0.06(1.08) | 0.446 | -0.29(1.07) | -0.09(1.02) | 0.367 |

† z-scores were generated by log-transformation of the original food intake, followed by sex standardisation based on the sex-specific distribution of the rural participants

*p-value for the effect of time in urban areas
